# Endophytic fungus *Biscogniauxia petrensis* produces antibacterial substances

**DOI:** 10.7717/peerj.15461

**Published:** 2023-06-07

**Authors:** Long Han, Wen Zheng, Zhangjiang He, Shengyan Qian, Xiaoya Ma, Jichuan Kang

**Affiliations:** 1 College of Life Sciences, Guizhou University, Guiyang, China; 2 Engineering Research Center of the Utilization for Characteristic Bio-pharmaceutical Resources in Southwestern, Ministry of Education, Guizhou University, Guiyang, China; 3 School of Science, Mae Fah Luang University, Chiang Rai, Thailand

**Keywords:** Medicinal plant, Endophytic fungus, Biscogniauxia petrensis, Foodborne pathogen, Antibacterial substance, Funicin, Vinetorin

## Abstract

Widespread drug resistance and limited antibiotics challenge the treatment of pathogenic bacteria, which leads to a focus on searching for new antimicrobial lead compounds. We found the endophytic fungus *Biscogniauxia petrensis* MFLUCC14-0151 from the medicinal plant *Dendrobium harveyanum* had antibacterial activity for the first time. This work aimed to reveal the capacity of *Biscogniauxia petrensis* MFLUCC14-0151 against foodborne pathogenic bacteria and identify its bioactive substances. Bioassay-guided isolation led to the discovery of six infrequent active monomers, including (10R)-Xylariterpenoid B (1), Xylariterpenoid C (2), Tricycloalternarene 1b (3), Tricycloalternarene 3b (4), Funicin (5) and Vinetorin (6) from MFLUCC14-0151 for the first time. The results of antibacterial tests showed that (10R)-Xylariterpenoid B and Xylariterpenoid C exhibited inhibitory activities against *Streptococcus agalactiae* with MIC values ranging from 99.21 to 100.00 μM, and against *Streptococcus aureus* with MIC values ranging from 49.60 to 50.00 μM. Tricycloalternarene 1b and Tricycloalternarene 3b showed inhibitory effects on *Streptococcus agalactiae* with MIC values ranging from 36.13 to 75.76 μM. Unexpectedly, Funicin and Vinetorin exhibited remarkable antagonistic activities against *Streptococcus agalactiae* with MIC values of 10.35 and 10.21 μM, respectively, and against *Streptococcus aureus* with MIC values of 5.17 and 20.42 μM, respectively. In conclusion, we suggest that the isolated compounds Funicin and Vinetorin may be promising lead compounds for natural antibacterial agents.

## Introduction

Endophytes are microorganisms living in the internal tissues of the host plants without causing any overt symptoms (Stone, Bacon & White, 2000). The host plants provide essential spaces and nutrients for endophytes, meanwhile the endophytes provide protection and survival value for host *via* producing various active substances. The medicinal plants were reported to be rich in endophytes with approximately one million species awaiting exploring (Gakuubi et al., 2021). Furthermore, the endophytes in medicinal plants possessed the ability to synthesize same or similar bioactive ingredients as those produced by the host (Prajapati, Goswami & Rawal, 2021). For example, the anti-tumor drugs paclitaxel, camptothecin, and the platelet activator inhibitor ginkgolide B, initially extracted from the medicinal plants have also been produced by their endophytes *viz* *Taxomyces andreanae*, *Paenibacillus polymyxa* and *Fusarium oxysporum*, respectively (Stierle, Strobel & Stierle, 1993; Pu et al., 2015; Cui et al., 2012). Searching for unique bioactive components from endophytes in unusual niches has important scientific and pharmaceutical values.

The genus *Biscogniauxia* belongs to the fungal family *Graphostromataceae*, and its species have been found mainly as pathogens in dicotyledonous plants worldwide (Evidente et al., 2005; Daranagama et al., 2018). Currently, numerous monomers with impressive chemical structures showing bioactivities of glycogen synthase kinase-inhibitory, acetylcholinesterase-inhibitory, anti-pathogenic fungi, anti-tumour and anti-Alzheimer’s disease (AD) activities, such as Isopyrrolonaphthoquinone, Biscogniphthalide, Eudesmanolide,Biscognienyne C, Biscognienyne D, Biscognienyne F and Dimericbiscognienyne A, have been isolated from this genus (Wu et al., 2016; Nguyen et al., 2018; Liu et al., 2019; Zhao et al., 2017, 2021a). However, there are few reports on the antibacterial activity of the *Biscogniauxia petrensis*.

In this study, *Biscogniauxia petrensis* MFLUCC14-0151 was firstly found to possess inhibitory effects on foodborne pathogenic bacteria *viz* *Streptococcus agalactiae* GBS-1, *Streptococcus aureus* SA-1 and *Escherichia coli* EC-1 with its crude extract of ethyl acetate. Meanwhile, six active compounds *viz* (10R)-Xylariterpenoid B (1), Xylariterpenoid C (2), Tricycloalternarene 1b (3), Tricycloalternarene 3b (4), Funicin (5) and Vinetorin (6) were isolated from the *Biscogniauxia* genus for the first time and the biological activities of them were originally reported.

## Materials and Methods

### Fungal and bacterial strains

*Biscogniauxia petrensis* MFLUCC14-0151 was isolated and identified by our research group (Ma et al., 2020), and deposited at the China General Microbiological Culture Collection Center (CGMCC 40341; No. 3, Yard 1, Beichen West Road, Chaoyang District, Beijing). The foodborne pathogenic bacteria *Streptococcus agalactiae* GBS-1, *Streptococcus aureus* SA-1 and *Escherichia coli* EC-1 were obtained from the Engineering Research Center of the Utilization for Characteristic Bio-pharmaceutical Resources in Southwestern, Ministry of Education, Guizhou University. The fungus strain was maintained at 4 °C on potato dextrose agar (PDA) and incubated at 28 °C on Martin modified (MM) medium. The tested pathogenic bacteria were cultivated in nutrient broth (NB) medium and nutrient agar (NA) medium at 37 °C.

### Strain cultivation and fermentation

The fungus strain was cultured on PDA at 28 °C for a week. Three pieces of mycelial agar plugs with a diameter 6 mm were inoculated into the Erlenmeyer flasks (500 mL), each containing 200 mL of Martin modified (MM) medium to obtain the seed culture. Fermentation was carried out in 250 Erlenmeyer flasks (volume 1 L), each containing 200 g of rice and 150 mL of distilled H_2_O, and then autoclaving at 120 °C for 30 min. Each flask was inoculated with 10 mL of seed culture and incubated at 28 °C under static condition for 2 months.

### Extract preparation

The crude extract was obtained using the method with some modifications (Wang et al., 2020). The whole cultures (60 kg) were extracted thrice with 120 L of methanol, and the organic solvent was evaporated to a small volume (5 L) under vacuum. The extract (900 g) was suspended in 10 L of distilled H_2_O and partitioned successively by extracting thrice with 2-fold volume of EtOAc (20 L) and n-BuOH (20 L), respectively. The EtOAc solution was evaporated under reduced pressure to obtain a crude extract (56 g).

### Antibacterial activity

The antagonistic activities of the crude extract against indicator bacteria *viz* *Streptococcus agalactiae* GBS-1, *Streptococcus aureus* SA-1 and *Escherichia coli* EC-1 strains were determined using agar diffusion method with some modifications (Ngamsurach & Praipipat, 2021). The analysis plates used in the assay were prepared *via* transferring 1 mL of each indicator bacterial suspension (10^8^ CFU/mL) into 100 mL NA medium (50–55 °C). The indicator bacteria and the medium were mixed homogeneously, and then the mixture was poured into a petri-dish (20 mL). The Oxford cups were added and the plate was left for approximately 2 h to solidify. The Oxford cups were removed, and followed by adding 10 μL of samples into the well and incubation for 24 h at 37 °C. The samples were prepared by dissolving the tested components in dimethyl sulfoxide (DMSO) to obtain a solution with a concentration of 2 mg/mL. Ampicillin (AMP) with a concentration of 500 μg/mL was used as the positive control, and DMSO as the negative control. All tests were performed in triplicates. The diameters of the inhibition zones were measured after 48 h incubation.

### Isolation and purification of active monomers

The crude extract (56 g) was subjected to silica gel column chromatography (CC) using EtOAc-MeOH (50:1, 25:1, 10:1, 5:1 and 1:1, v/v) to yield six fractions: A1, A2, A3, A4, A5 and A6. The fractions were further used for the determination of antibacterial activity. The main bioactive fractions *viz* A3, A4 and A5 were further separated and purified. The fraction A3 (7.454 g) was subjected to silica gel CC using PE-EtOAc (20:1, 10:1, 1:1, v/v) and pure MeOH to yield nine sub-fractions A3-(1-9). The bioactive fraction A3-7 (1.2 g) was further subjected to ODS MPLC (4 cm × 45 cm) eluted with MeOH-H_2_O (80:20, v/v) to yield six sub-fractions A3-7(1-6). The bioactive fraction A3-7-4 (158 mg) was purified *via* silica gel CC using PE-EtOAc (5:1, v/v) to elute and yielded compound 5 (5 mg), compound 3 (13 mg) and compound 4 (17 mg).

The fraction A4 (6.815 g) was separated by ODS MPLC (4 cm × 40 cm) eluted with MeOH-H_2_O (20:80, 40:60, 60:40, and 100:0, v/v) to yield six sub-fractions A4 (1-6). The bioactive fraction A4-2 (2.26 g) was further subjected to PE-AC (5:1, v/v) to obtain bioactive fraction A4-2-3. The fraction A4-2-3 (188 mg) was subjected to ODS MPLC (2.5 cm × 20 cm) eluted with MeOH-H_2_O (40:60, v/v) to yield compound 6 (20 mg).

The fraction A5 (7.120 g) was separated into nine sub-fractions A5 (1-9) using ODS MPLC (3 cm × 75 cm) eluted with MeOH-H_2_O (20:80, 40:60, 80:20 and 100:0, v/v) to obtain bioactive fraction A5-2. The fraction A5-2 (498 mg) was further subjected to silica gel CC eluted with PE-AC (3:1, v/v) to yield compound 2 (40 mg) and compound 1 (10 mg).

### Structure elucidation of monomers

A total of 5 mg of compounds were dissolved in 600 μL of deuterium reagent in the NMR tubes, and then the ^1^H NMR and ^13^C NMR spectra were recorded on a Bruker 500 MHz NMR apparatus (Bruker, Bremerhaven, Germany). The spectra data were processed and analyzed with MestReNova-9.0.1 software. The known compounds were identified by comparing its nuclear magnetic resonance spectral data (^1^H-NMR and ^13^C-NMR) with those of compounds reported in NMR Carbon Spectrum Database of Organic Compounds (http://www.nmrdata.com).

### Determination of the minimum inhibitory concentration

The minimum inhibitory concentration (MIC) measurement was conducted to evaluate the antibacterial activities of compounds **1**-**6** against three tested pathogenic bacteria (GBS-1, SA-1 and EC-1 strains) *via* broth microdilution method with some modifications (Chen et al., 2019). The monomers were dissolved in DMSO (1 mg/mL), and then the solution was diluted at concentrations of (500, 250, 125, 62.5, 31.3, 15.6, 7.81, 3.91, 1.95, 0.98 µg/mL) by twofold dilutions in a 96-well plate. The tested bacterial suspension was adjusted to a density of bacterial cells of 10^6^ CFU/mL. To each well, 100 µL of each bacterial suspension was inoculated and incubated at 37 °C for 24 h. The MIC values were recorded as the lowest concentrations of the monomers that had no visible bacterial turbidity according to the guidelines (M27-A3) (CLSI 2008) (Iraji et al., 2020). The tests using DMSO as negative control and Ampicillin (AMP) as positive control were carried out in parallel. Each treatment was repeated three times.

### Statistical analysis

All experiments were conducted in three times and expressed as mean ± standard deviation. The data was analyzed using a t test and the differences among groups were evaluated by Two-way analyses of variance (ANOVA) using the GraphPad Prism software (version 5; GraphPad, San Diego, CA, USA). *P* < 0.05 and *P* < 0.01 were considered significant and highly significant differences, respectively.

## Results

### Antibacterial activities of crude extract

**Figure 1:**
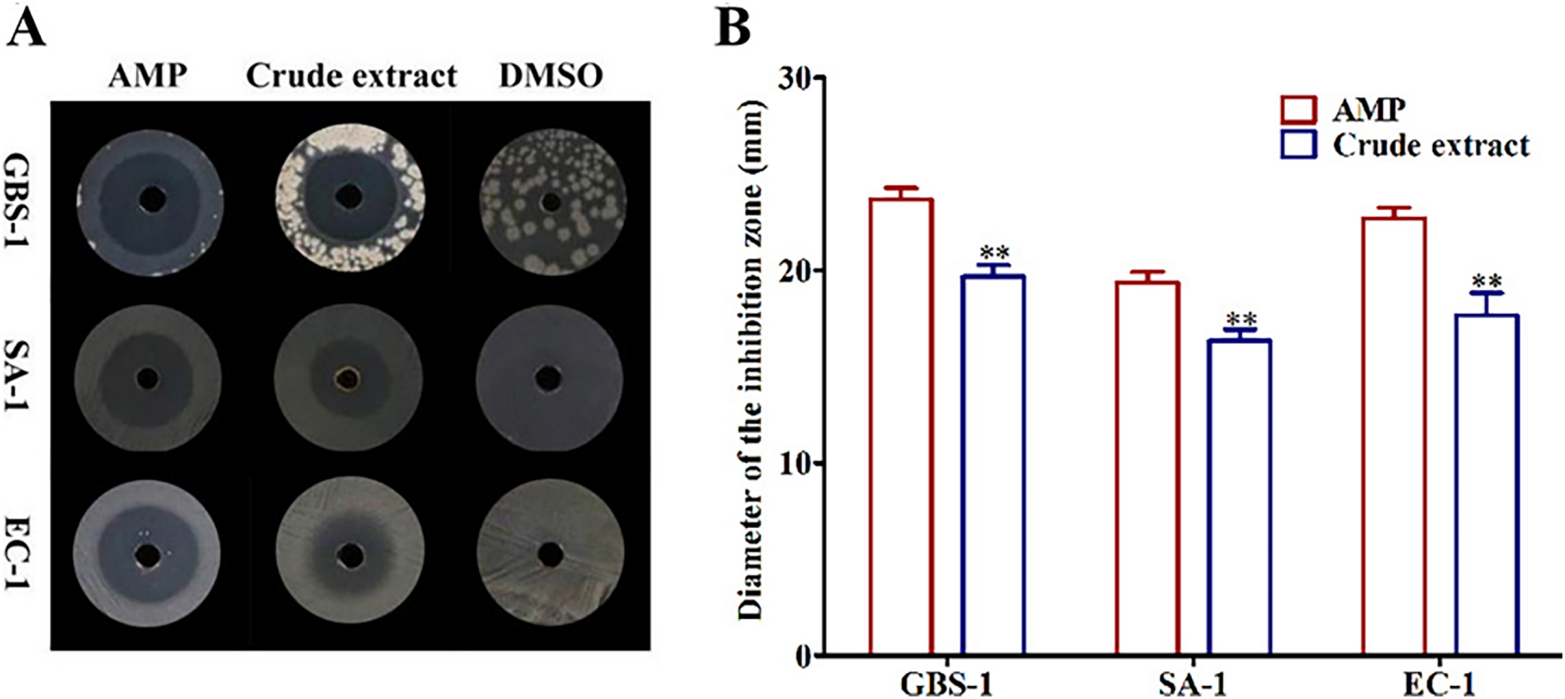
The crude extract of ethyl acetate from MFLUCC14-0151 showed antagonistic activities against test pathogenic bacteria. (A) The inhibition zones of AMP, crude extract and DMSO against test bacteria. (B) The diameters of inhibition zones of AMP and crude extract against test bacteria. AMP was positive control and DMSO was negative control. GBS-1, SA-1 and EC-1 represented *S*. *agalactiae* GBS-1, *S. aureus* SA-1 and *E. coli* EC-1, respectively. Asterisks (**) indicated significant differences (*P* < 0.05).

In order to determine whether the crude extract showed antagonistic activities against foodborne pathogenic bacteria, *Streptococcus agalactiae* GBS-1, *Streptococcus aureus* SA-1 and *Escherichia coli* EC-1 were used as tested strains to detect the inhibitory effects of the crude extract on their growth. We observed that the crude extract had obvious bacteriostatic zones against all tested pathogenic bacteria contrasted with the negative control DMSO (Fig. 1A). The inhibitory activities of the crude extract against three tested bacteria were GBS-1 > EC-1 > SA-1 with the diameter of inhibition zones were 19.67, 17.67 and 16.33 mm, respectively, but none of them were as strong as the positive control AMP (*P* < 0.05) (Fig. 1B). It indicated that *Biscogniauxia petrensis* MFLUCC14-0151 produced one or more antibacterial active ingredients.

### Three sub-fractions showed antagonistic activities

**Figure 2:**
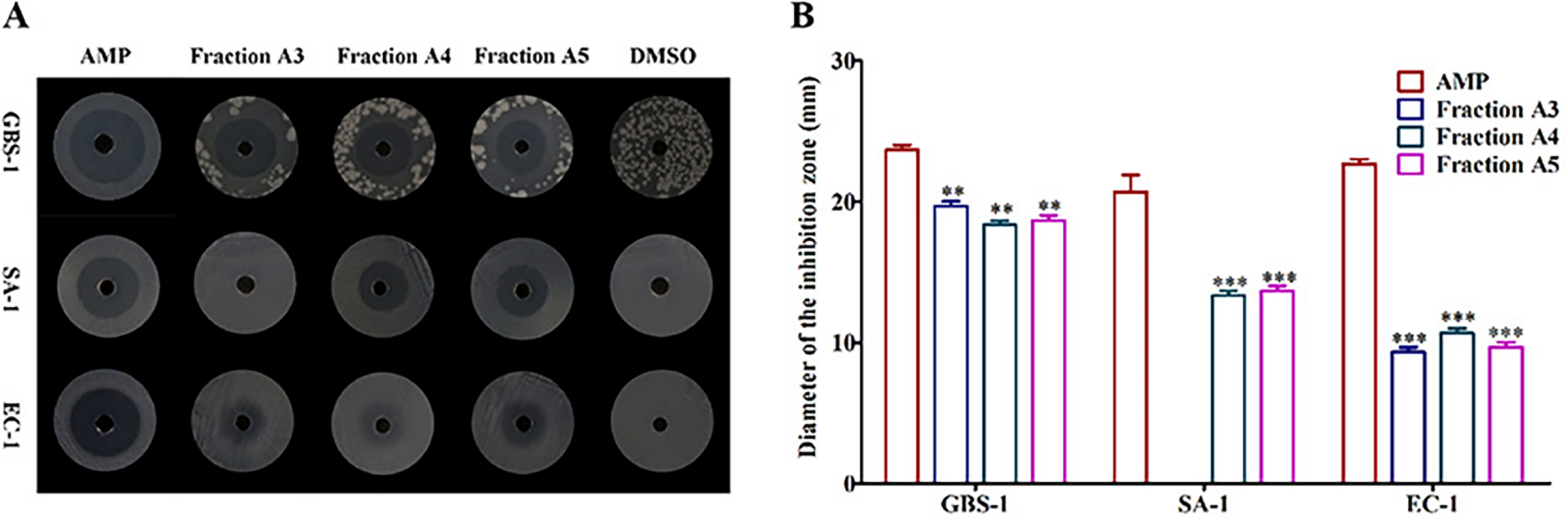
The fractions A3, A4 and A5 showed antagonistic activities against test pathogenic bacteria. (A) The inhibition zones of AMP, fractions A3-A5 and DMSO against test bacteria. (B) The diameters of inhibition zones of AMP and fractions A3-A5 against test bacteria. AMP was positive control and DMSO was negative control. GBS-1, SA-1 and EC-1 represented *S. agalactiae* GBS-1, *S. aureus* SA-1 and *E. coli* EC-1, respectively. Asterisks (** and ***) indicated significant differences (*P* < 0.05) and highly significant differences (*P* < 0.01), respectively.

To determine the distributions of active constituents against tested pathogenic bacteria, the crude extract was divided into different fractions and their bioactivities were tracked. The antibacterial activities of six fractions (A1-A6) obtained from the crude extract were tested. The results suggested that fractions A4 and A5 had antagonistic activities against all tested pathogenic bacteria contrasted with the negative control DMSO (Fig. 2A), but their inhibitory activities were far less than those of positive control AMP (*P* < 0.05 and *P* < 0.01) (Fig. 2B). The fraction A3 performed inhibitory effects on *Streptococcus agalactiae* GBS-1 and *Escherichia* EC-1, without *Streptococcus aureus* SA-1 (*P* < 0.05 and *P* < 0.01) (Figs. 2A and 2B). Those results confirmed that diverse active substances produced by *Biscogniauxia petrensis* MFLUCC14-0151, and provided transparent guidance for the subsequent targeted separation and purification of active monomers.

### Structure elucidation of monomeric compounds

**Figure 3:**
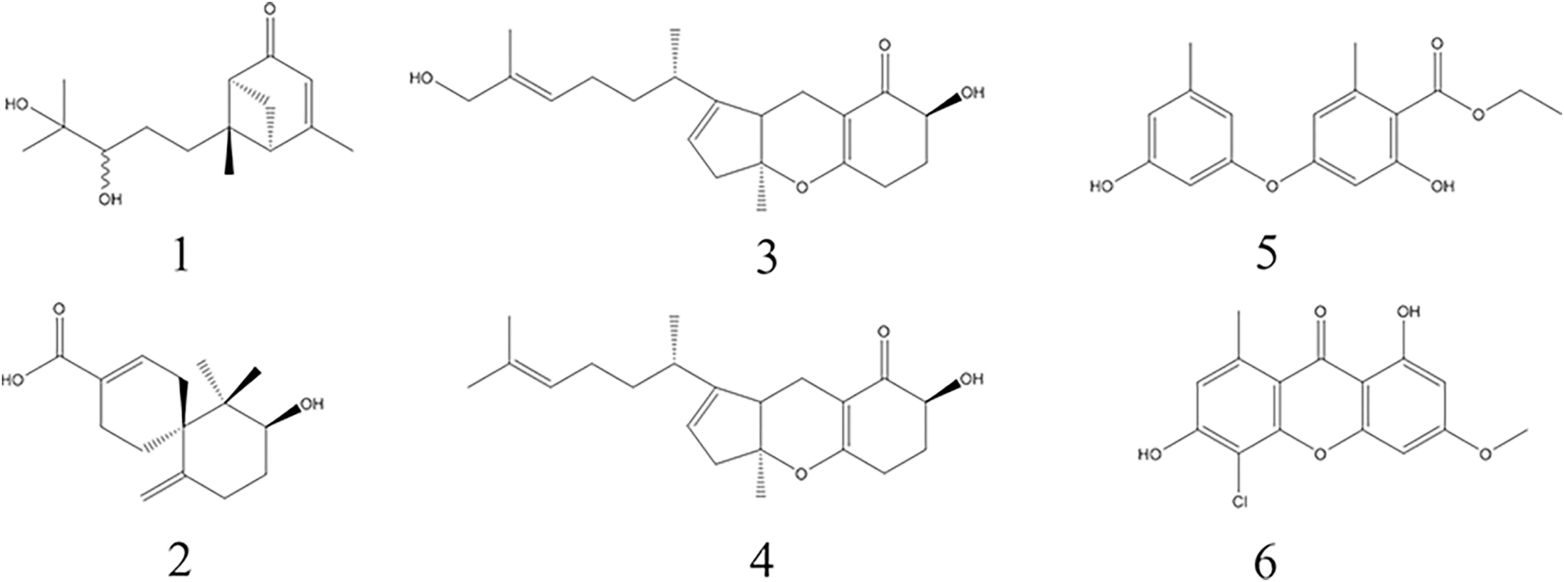
The chemical structures of the six monomers isolated from MFLUCC14-0151. Compounds 1-6 are (10R)-Xylariterpenoid B, Xylariterpenoid C, Tricycloalternarene 1b, Tricycloalternarene 3b, Funicin and Vinetorin, respectively.

Six known monomers were separated from the three bioactive fractions A3-A5, respectively. Their chemical structures were confirmed by comparing with the NMR spectroscopy (^1^H-NMR and ^13^C-NMR) data reported in previous literature. They were identified as (10R)-Xylariterpenoid B (1), Xylariterpenoid C (2), Tricycloalternarene 1b (3), Tricycloalternarene 3b (4), Funicin (5) and Vinetorin (6) (Fig. 3).

Compound 1: colorless solid, ^1^H NMR (500 MHz, CD_3_OD) δ: 5.74 (1H, s, H-4), 3.26 (1H, dd, *J* = 10.1, 1.75 Hz, H-10), 2.90 (1H, dt, *J* = 9.3, 5.5 Hz, H-1), 2.67 (1H, td, *J* = 6.25, 1.7 Hz, H-6), 2.59 (1H, td, *J* = 6.5, 1.3 Hz, H-2), 2.24 (1H, td, *J* = 13, 4.4 Hz, H-8a), 2.09 (1H, d, *J* = 9.3 Hz, H-1), 2.06 (3H, d, *J* = 1.6 Hz, H-15), 1.89 (1H, dt, *J* = 12.1, 4.7 Hz, H-8b), 1.71 (1H, m, H-9a), 1.33 (1H, m, H-9b), 1.20 (3H, s, H-12), 1.16 (3H, s, H-13), 1.00 (3H, s, H-14); ^13^C NMR (125 MHz, CD_3_OD) δ: 207.0 (s, C-5), 174.3 (s, C-3), 122.0 (s, C-4), 80.0 (s, C-10), 73.9 (s, C-11), 58.9 (s, C-7), 57.4 (s, C-6), 49.7 (s, C-2), 42.0 (s, C-1), 37.0 (s,C-8), 27.1 (s, C-9), 26.1 (s, C-12), 24.6 (s, C-13), 23.7 (s, C-15), 19.4 (s, C-14). The spectral data of compound 1 were in agreement with the data previously reported for (10R)-Xylariterpenoid B (Niu et al., 2017). Hence, compound 1 was confirmed as (10R)-Xylariterpenoid B (Fig. S1).

Compound 2: colorless solid, ^1^H NMR (500 MHz, CDCl_3_) δ: 7.09 (1H, m, H-2), 4.91 (1H, s, H-14b), 4.44 (1H, s, H-14a), 3.89 (1H, dd, *J* = 11.9, 4.7 Hz, H-10), 2.32 (3H, m, H-1b, H-4b, H-8b), 2.17 (1H, m, H-8a), 1.95 (1H, m, H-5b), 1.86 (1H, m, H-9b), 1.78 (1H, m, H-4a), 1.48 (1H, m, H-9a), 1.46 (1H, m, H-9a), 1.01 (3H, s, H-13), 0.75 (3H, s, H-12); ^13^C NMR (125 MHz, CDCl_3_) δ: 172.0 (s, C-15), 146.4 (s, C-7), 141.5 (s, C-2), 128.9 (s, C-3), 112.1 (s, C-14), 73.3 (s, C-10), 45.4 (s, C-6), 41.9 (s, C-11), 31.9 (s, C-9), 30.2 (s, C-1), 30.0 (s, C-8), 25.2 (s, C-5), 21.5 (s, C-4), 20.4 (s, C-13), 15.1 (s, C-12). Comparing with the spectral data in the literature (Wu et al., 2014), compound 2 was confirmed as Xylariterpenoid C (Fig. S2).

Compound 3: colorless solid, ^1^H NMR (500 MHz, CDCl_3_) δ: 5.28 (1H, s br, H-8), 3.99 (1H, dd, *J* = 12.7, 6.7 Hz, H-17), 3.40 (1H, m, H-1a), 3.35 (1H, m, H-1b), 2.54 (1H, s, H-9b), 2.42 (1H, m, H-15a and H-9a), 2.36 (1H, m, H-15b), 2.31 (1H, m, H-16b), 2.17 (1H, m, H-12a), 1.96 (1H, m, H-6), 1.70 (1H, m, H-16a), 1.53 (1H, m, H-2), 1.40 (4H, s, H-10′ and H-5a), 1.28 (1H, m, H-3a), 1.23 (1H, m, H-4a), 1.19 (1H, m, H-5b), 1.17 (1H, m, H-4b), 0.97 (1H, m, H-3b), 0.93 (3H, d, *J* = 11.6 Hz, H-6′), 0.86 (3H, d, *J* = 8.4 Hz, H-2′); ^13^C NMR (125 MHz, CDCl_3_) δ: 197.8 (s, C-18), 172.5 (s, C-14), 150.4 (s, C-7), 119.8 (s, C-8), 105.2 (s, C-13), 88.4 (s, C-10), 71.0 (s, C-17), 68.3 (s, C-1), 46.4 (s, C-11), 44.9 (s, C-9), 35.5 (s, C-2), 35.1 (s, C-5), 33.1 (s, C-3), 32.5 (s, C-6), 29.5 (s, C-16), 27.8 (s, C-15), 24.6 (s, C-4), 23.3 (s, C-10′), 20.2 (s, C-6′), 16.6 (s, C-2′), 15.3 (s, C-12). The spectral data of compound 3 were consistent with the data of Tricycloalternarene 1b (Liebermann et al., 1997). Therefore, compound 3 was confirmed as Tricycloalternarene 1b (Fig. S3).

Compound 4: colorless solid, ^1^H NMR (500 MHz, CDCl_3_) δ: 5.32 (1H, br s, H-11), 5.03 (1H, m, H-16), 4.03 (1H, dd, *J* = 13.0, 5.3 Hz, H-2), 2.74 (1H, br s, H-8), 2.67 (1H, m, H-7a), 2.61 (1H, br s, H-10a), 2.49~2.46 (1H, m, H-10b), 2.39 (1H, m, H-4a), 2.34~2.29 (2H, m, H-4b, H-7b), 2.23 (1H, m, H-3a), 2.01 (1H, m, H-13), 1.88 (2H, s, H-15), 1.66 (3H, m, H-18), 1.60 (1H, m, H-3b), 1.55 (3H, s, H-21), 1.48 (1H, m, H-14a), 1.43 (3H, s, H-19), 1.28 (1H, m, H-14b), 0.96 (3H, d, *J* = 5 Hz, H-20); ^13^C NMR (125 MHz, CDCl_3_) δ: 197.8 (s, C-1), 172.5 (s, C-5), 150.4 (s, C-12), 131.4 (s, C-17), 124.4 (s, C-16), 119.8 (s, C-11), 105.2 (s, C-6), 88.4 (s, C-9), 71.0 (s, C-2), 46.4 (s, C-8), 44.9 (s, C-10), 35.5 (s, C-14), 32.1 (s, C-13), 29.5 (s, C-3), 27.8 (s, C-4), 25.9 (s, C-15), 25.7 (s, C-18), 23.4 (s, C-19), 20.2 (s, C-20), 17.7 (s, C-21), 15.4 (s, C-7). The spectral data of compound 4 were in accordance with the data previously reported for Tricycloalternarene 3b (Yoiprommarat et al., 2015). Hence, compound 4 was confirmed as Tricycloalternarene 3b (Fig. S4).

Compound 5: white powder, ^1^H NMR (500 MHz, CDCl_3_) δ: 11.74 (1H, s, 3-OH), 6.48 (1H, m, H-4′), 6.45 (1H, m, H-6′), 6.36 (1H, m, H-2′), 6.34 (1H, m, H-2), 4.42 (2H, q, *J* = 7.15 Hz, H-9), 2.51 (3H, s, H-7), 2.29 (3H, s, H-7′), 1.42 (3H, t, *J* = 7.15 Hz, H-10); ^13^C NMR (125 MHz, CDCl_3_) δ: 171.6 (s, C-8), 165.1 (s, C-3), 162.2 (s, C-1), 156.6 (s, C-5′), 156.0 (s, C-1′), 143.7 (s, C-5), 141.3 (s, C-3′), 113.6 (s, C-2′), 113.0 (s, C-6′), 112.5 (s, C-4′), 107.1 (s, C-4), 105.0 (s, C-6′), 103.3 (s, C-2), 61.5 (s, C-9), 24.4 (s, C-7), 21.5 (s, C-7′), 14.3 (s, C-10). The spectral data of compound 5 showed no difference with that reported in the literature for Funicin (Hamasaki et al., 1980). Therefore, compound 5 was confirmed as Funicin (Fig. S5).

Compound 6: white powder, ^1^H NMR (500 MHz, DMSO-*d*_*6*_) δ: 13.2 (1H, s, 1-OH), 11.65 (1H, br s, 6-OH), 6.8 (1H, d, *J* = 0.8 Hz, H-7), 6.5 (1H, d, *J* = 2.3 Hz, H-4), 6.3 (1H, d, *J* = 2.3 Hz, H-2), 3.9 (3H, s, 3-OMe), 2.7 (3H, d, 8-Me); ^13^C NMR (125 MHz, DMSO-*d*_*6*_) δ: 181.8 (s, C-9), 166.3 (s, C-3), 163.1 (s, C-1), 159.2 (s, C-6), 156.2 (s, C-4a), 154.4 (s, C-10a), 140.9 (s, C-8), 116.0 (s, C-7), 112.2 (s, C-8a), 105.1 (s, C-5), 103.3 (s, C-9a), 97.9 (s, C-2), 92.6 (s, C-4), 56.6 (s, 3-OMe), 23.4 (s, 8-Me). Comparison with its spectral data in the literature (Hu, Yip & Sim, 1999), compound 6 was confirmed as Vinetorin (Fig. S6).

### Monomers showed antibacterial activities

To evaluate the antibacterial potential of monomers against pathogenic bacteria. The minimum inhibitory concentrations (MICs) of compounds 1-6 against *Streptococcus agalactiae* GBS-1, *Streptococcus aureus* SA-1 and *Escherichia coli* EC-1 were determined. The results showed that all monomers exhibited different antagonistic activities (Table 1). In comparison with other compounds, Funicin exhibited stronger inhibitory effects on *Streptococcus agalactiae* GBS-1 and *Streptococcus aureus* SA-1 with MIC values of 10.35 and 5.17 μM, respectively, followed by Vinetorin with MIC values of 10.21 and 20.42 μM, respectively. (10R)-Xylariterpenoid B and Xylariterpenoid C showed inhibitory activities against *Streptococcus agalactiae* GBS-1 and *Streptococcus aureus* SA-1 with MIC values in the range of 49.60–100 μM. Tricycloalternarene 1b manifested stronger antibacterial activity with MIC value of 36.13 μM than Tricycloalternarene 3b with MIC value of 75.76 μM against *Streptococcus agalactiae* GBS-1, while they exhibited no inhibitory effect on *Streptococcus aureus* SA-1 with MIC value >150 μM. For the *Escherichia coli* EC-1, (10R)-Xylariterpenoid B displayed stronger activity with MIC value of 49.60 μM than that of Funicin with MIC value of 82.78 μM, while all others exhibited no inhibitory effect with MIC > 150 μM. Taken together, the tested monomers were found to possess different antimicrobial activities against diverse pathogenic bacteria, among which Funicin and Vinetorin demonstrated stronger antibacterial effects.

**Table 1:** The minimum inhibitory concentration (MIC, µM) of the monomeric compounds.

Compounds	GBS-1	SA-1	EC-1
(10R)-Xylariterpenoid B (**1**)	99.21	49.60	49.60
Xylariterpenoid C (**2**)	100.00	50.00	200.00
Tricycloalternarene 1b (**3**)	36.13	>200.00	>200.00
Tricycloalternarene 3b (**4**)	75.76	>151.52	>151.52
Funicin (**5**)	10.35	5.17	82.78
Vinetorin (**6**)	10.21	20.42	>163.40
AMP	2.23	1.12	1.12

**Note:**

AMP, Ampicillin is the positive.

## Discussion

Although the fungi in *Biscogniauxia* genus are generally regarded as plant pathogenic fungi, they might be endophytic fungi before transforming into pathogen microorganisms in the host, and possess the potential to synthesize and metabolize active constituents which are similar with those produced by the host plant in activity or structure. The metabolites derived from *Biscogniauxia* fungi had multiple bioactivities, such as anti-fungal, anti-inflammatory, anti-cancer and anti-Alzheimer’s disease (Jantaharn et al., 2021; Zhao et al., 2021b). The antibacterial activity of *Biscogniauxia* was rarely reported. In our study, *Biscogniauxia petrensis* MFLUCC14-0151 from the medicinal plant *Dendrobium harveyanum* was found to repress foodborne pathogenic bacteria *Streptococcus agalactiae* GBS-1, *Streptococcus aureus* SA-1, and *Escherichia coli* EC-1 for the first time. Furthermore, the active substances were isolated, purified and identified as (10R)-Xylariterpenoid B, Xylariterpenoid C, Tricycloalternarene 1b, Tricycloalternarene 3b, Funicin and Vinetorin.

As far as we know, the six scarce monomers were isolated from *Biscogniauxia* genus for the first time and their biological activities were originally reported. The (10R)-Xylariterpenoid B, a sesquiterpene, was only discovered in the marine fungus *Graphostroma* sp. MCCC 3A00421, which had weak anti-inflammatory effect (Niu et al., 2017). Our results showed that (10R)-Xylariterpenoid B had weak antagonistic activity against *Streptococcus agalactiae* GBS-1 and moderate inhibitory effect on *Streptococcus aureus* SA-1 and *Escherichia coli* EC-1. Xylariterpenoid C, a sesquiterpene, which was only isolated from *Xylariaceae* fungus, exhibited no significant cytotoxicity (Wu et al., 2014). However, we found that Xylariterpenoid C had weak and moderate antagonistic activities against *Streptococcus agalactiae* GBS-1 and *Streptococcus aureus* SA-1, respectively. Tricycloalternarene 1b and Tricycloalternarene 3b are mixed terpenoids, which were initially discovered in *Alternaria alternata* (Liebermann et al., 1997). Tricycloalternarene 1b and Tricycloalternarene 3b showed anti-tumor effects on mouse Lewis lung carcinoma cells 3LL and human neuroblastoma SH-SY5Y. (Yuan, Huang & Zhao, 2013). In addition, the compounds of Tricycloalternarene type have also been discovered in fungi *Septoria* sp., *Ulocladium* sp., *Aspergillus* sp., *Colletotrichum capsici* and *Didymella* sp. (Sugawara et al., 1998; Wang et al., 2013, 2016; Zhang et al., 2018; Li et al., 2018). The antibacterial tests indicated that Tricycloalternarene 1b and Tricycloalternarene 3b had antagonistic activities against *Streptococcus agalactiae* GBS-1. Funicin, a diphenyl ether substance, was previously discovered in *Aspergillus* sp. with remarkable antimicrobial activities against *Trichophyton asteroides*, *Trichophyton rubrum*, and *Trichophyton interdigitale* (Hamasaki et al., 1980). In the study, Funicin displayed stronger inhibitory activities against *Streptococcus agalactiae* GBS-1 and *Streptococcus aureus* SA-1 with MIC values ranging from 5.17 to 10.35 μM. Vinetorin, a xanthone containing chlorine atoms, was discovered in lichen and higher plant *Hypericum ascyron*, but its biological activity has not been reported (Feige & Lumbsch, 1993; Hu, Yip & Sim, 1999). Our data revealed that Vinetorin presented significant inhibition effects on *Streptococcus agalactiae* GBS-1 and *Streptococcus aureus* SA-1 with MIC values ranging from 10.21 to 20.42 μM. In general, Funicin and Vinetorin were main bioactive substances and can be used as lead molecules of antimicrobial agents for foodborne pathogens. Using Funicin and Vinetorin as prodrugs, their bioactivities and yields are improved, and their toxicities are weakened *via* chemical modification. Meanwhile, it is awaiting revealing the antibacterial mechanism of these active compounds.

## Conclusions

In summary, we found for the first time that the endophytic fungus *Biscogniauxia petrensis* exhibited antibacterial activity. Six infrequent monomers were isolated from the *Biscogniauxia petrensis* and their chemical structures were determined by spectroscopic analyses. Funicin and Vinetorin were the main bioactive substances and showed inhibitory effects on *Streptococcus agalactiae* and *Streptococcus aureus* with MIC values ranging from 5.17 to 20.42 μM. These data confirmed that the endophytic fungus *B. petrensis* is a new source of antibacterial substances, and Funicin and Vinetorin can be considered as lead compounds of antibacterial inhibitors.

## Supplemental Information

10.7717/peerj.15461/supp-1 Supplemental Information 1 The raw data for diameter of inhibition zone.

10.7717/peerj.15461/supp-2 Supplemental Information 2 NMR data and original figures of MIC of compounds (1-6).

## Additional Information and Declarations

### Data Availability

The following information was supplied regarding data availability: The raw measurements are available as a Supplemental File. Biscogniauxia petrensis MFLUCC14-0151 was deposited at the China General Microbiological Culture Collection Center (CGMCC 40341; No. 3, Yard 1, Beichen West Road, Chaoyang District, Beijing).
