# Supplementary material for: Endophytic fungus Biscogniauxia petrensis produces antibacterial substances
Source: PeerJ. 2023 Jun 7;11:e15461. doi: 10.7717/peerj.15461 (PMC10257390; doi:10.7717/peerj.15461)
Supplement: Supplemental Information 1 [file peerj-11-15461-s001.docx]

The raw data for diameter inhibition zone of the crude extract (Figure **1**)

| Strains | Crude extract | Ampicillin*^a^* | DMSO |
| --- | --- | --- | --- |
|  | Diameter of inhibition zone (mm) | | |
| GBS-1 | 20 | 24 | 0 |
|  | 20 | 23 | 0 |
|  | 19 | 24 | 0 |
| SA-1 | 16 | 20 | 0 |
|  | 16 | 23 | 0 |
|  | 17 | 19 | 0 |
| EC-1 | 19 | 22 | 0 |
|  | 17 | 23 | 0 |
|  | 17 | 23 | 0 |

*^a^*Ampicillin: the positive control

The raw data for diameter inhibition zone of three active fractions A3-A (Figure **2**)

| Strains | A3 | A4 | A5 | Ampicillin*^a^* | DMSO |
| --- | --- | --- | --- | --- | --- |
|  | Diameter of inhibition zone (mm) | | | | |
| GBS-1 | 20 | 18 | 19 | 24 | 0 |
|  | 20 | 18 | 18 | 23 | 0 |
|  | 19 | 19 | 19 | 24 | 0 |
| SA-1 | 0 | 13 | 14 | 23 | 0 |
|  | 0 | 13 | 13 | 20 | 0 |
|  | 0 | 14 | 14 | 19 | 0 |
| EC-1 | 9 | 10 | 10 | 22 | 0 |
|  | 10 | 11 | 10 | 23 | 0 |
|  | 9 | 11 | 9 | 23 | 0 |

*^a^*Ampicillin: the positive control
