# Supplementary material for: Endophytic fungus Biscogniauxia petrensis produces antibacterial substances
Source: PeerJ. 2023 Jun 7;11:e15461. doi: 10.7717/peerj.15461 (PMC10257390; doi:10.7717/peerj.15461)

Supplementary Figure 1. The NMR data of compound 1 in CD_3_OD (500 MHz for ^1^ H NMR, 125 MHz for ^13^ C NMR)


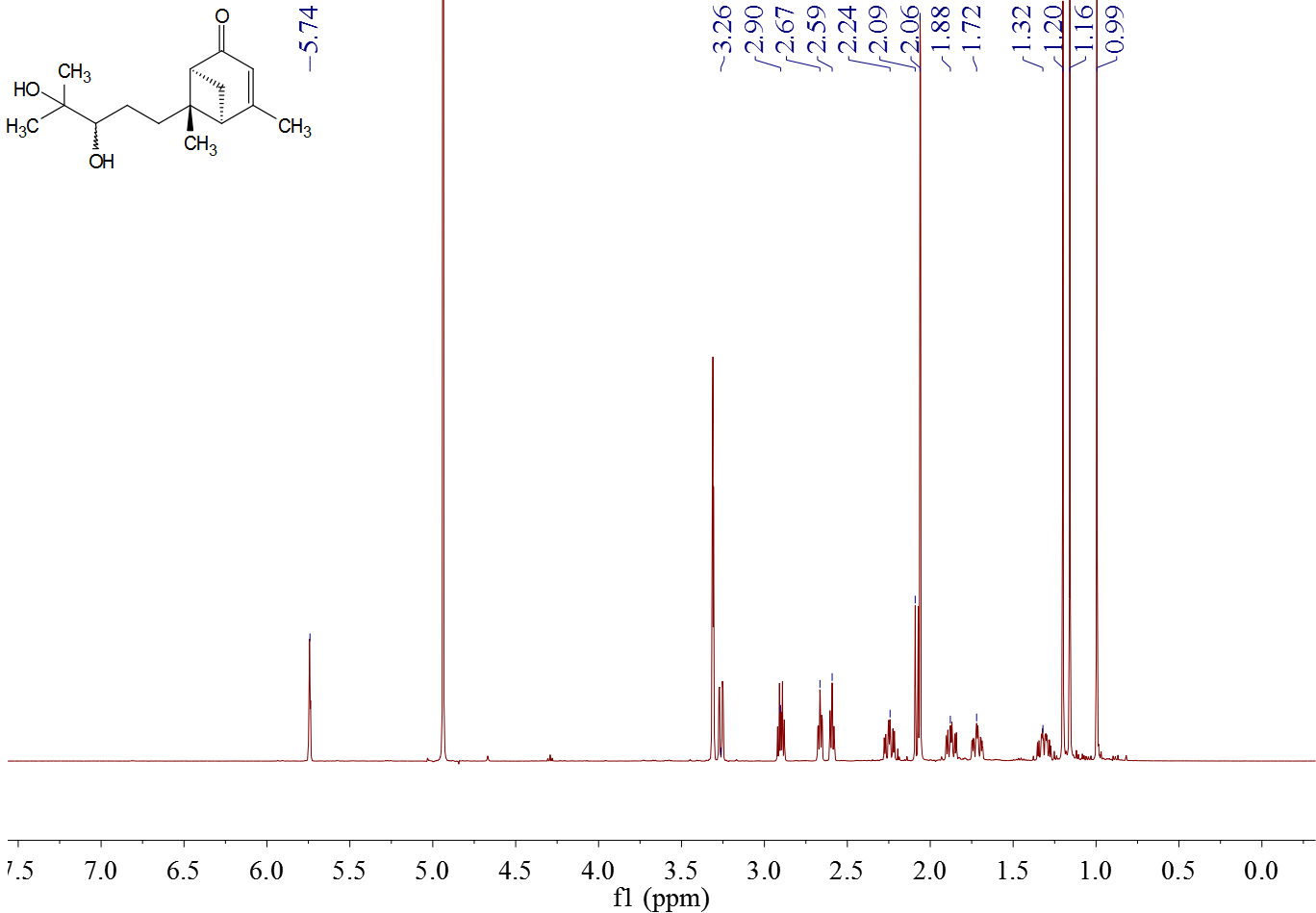


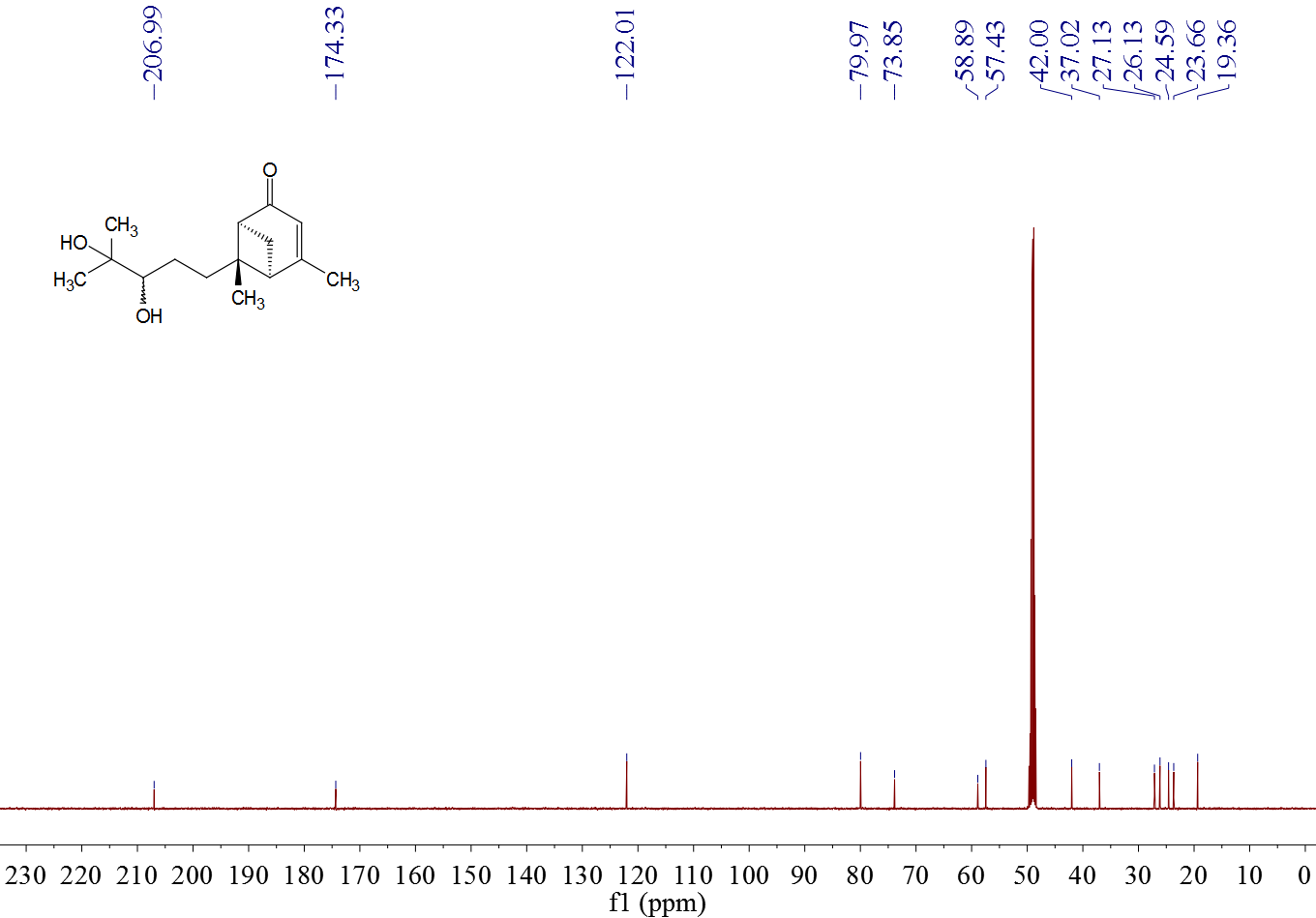


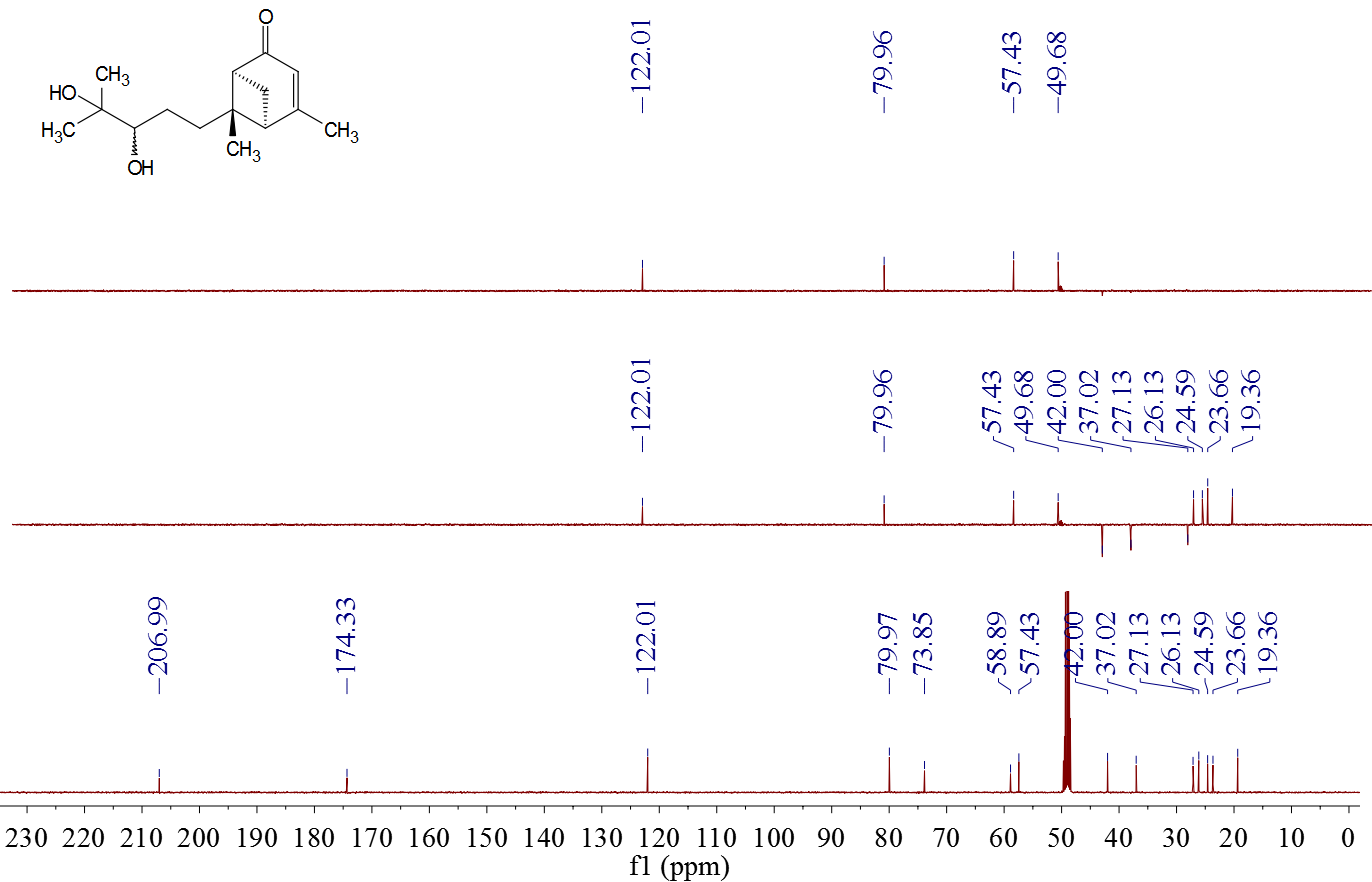


Supplementary Figure 2. The NMR data of compound 2 in CDCl_3_ (500 MHz for ^1^ H NMR, 125 MHz for ^13^ C NMR)


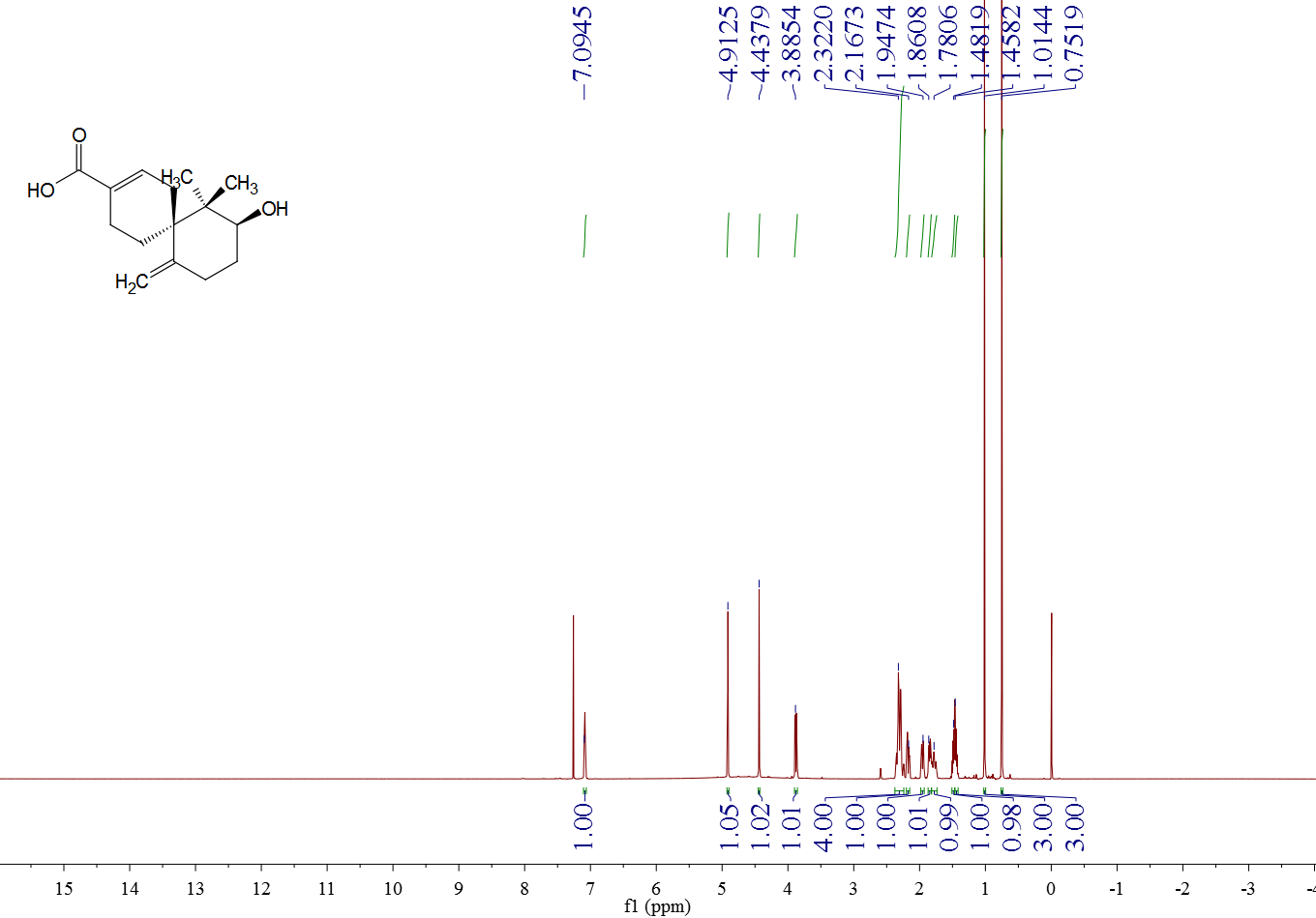


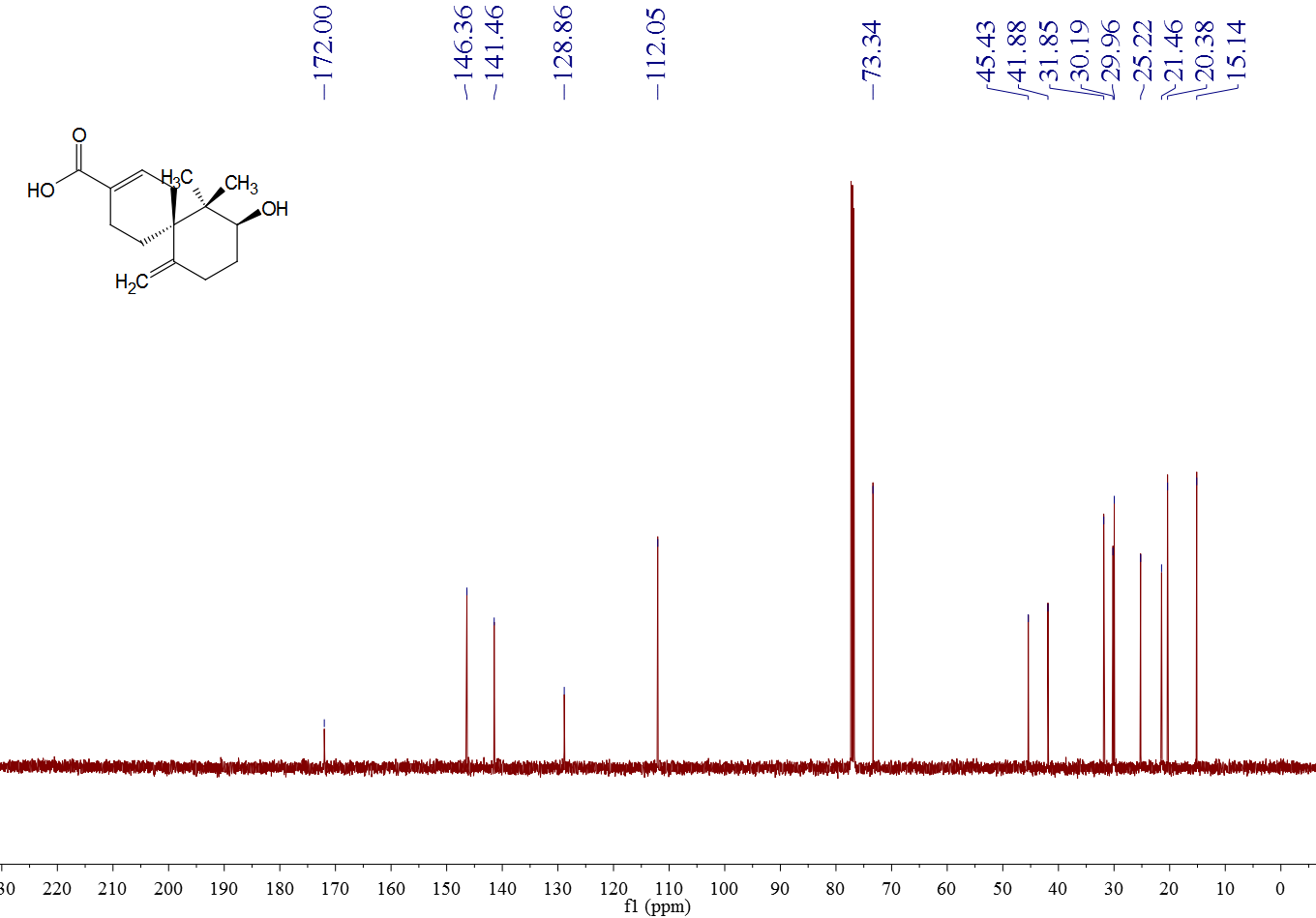


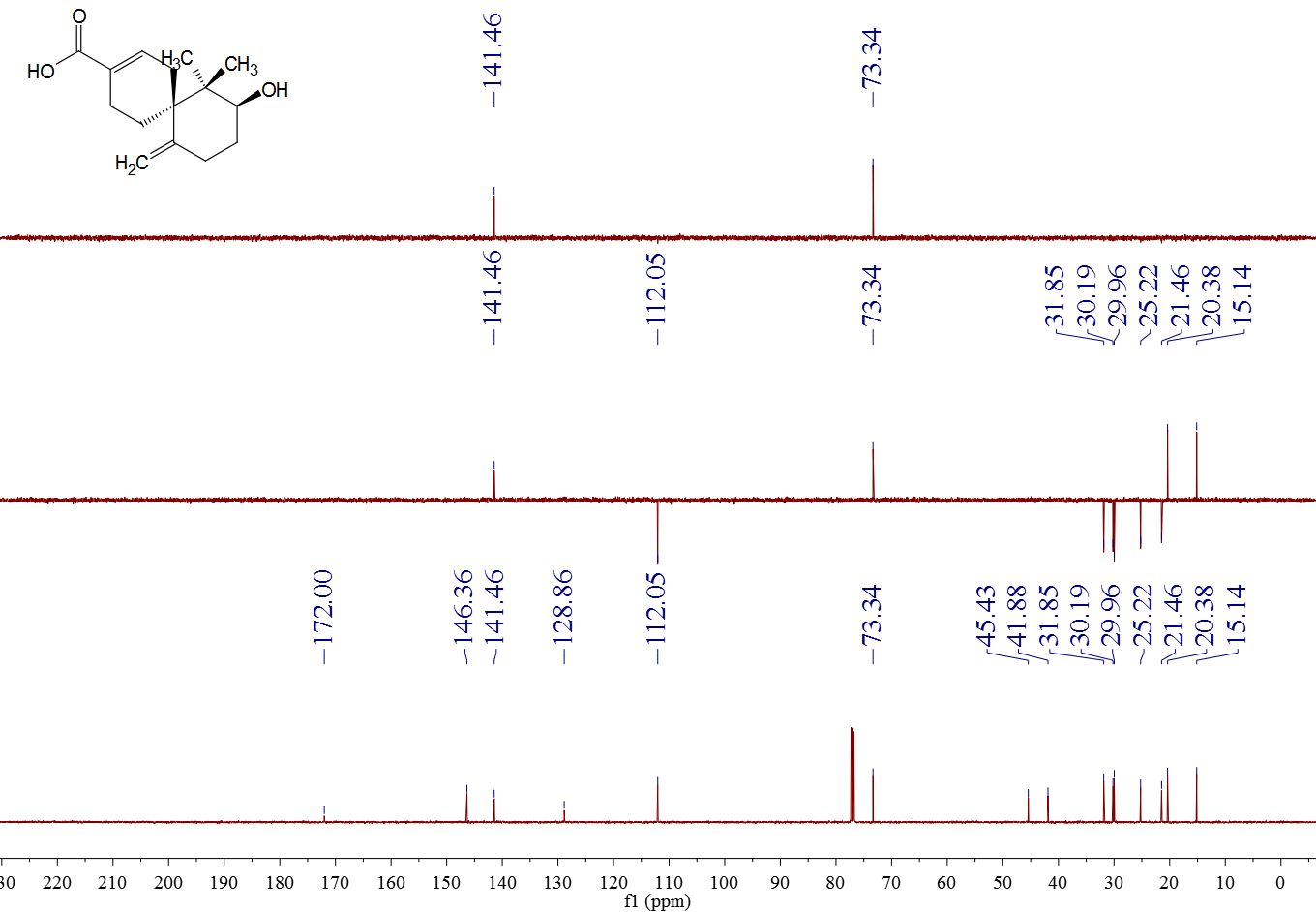


Supplementary Figure 3. The NMR data of compound 3 in CDCl_3_ (500 MHz for ^1^ H NMR, 125 MHz for ^13^ C NMR)


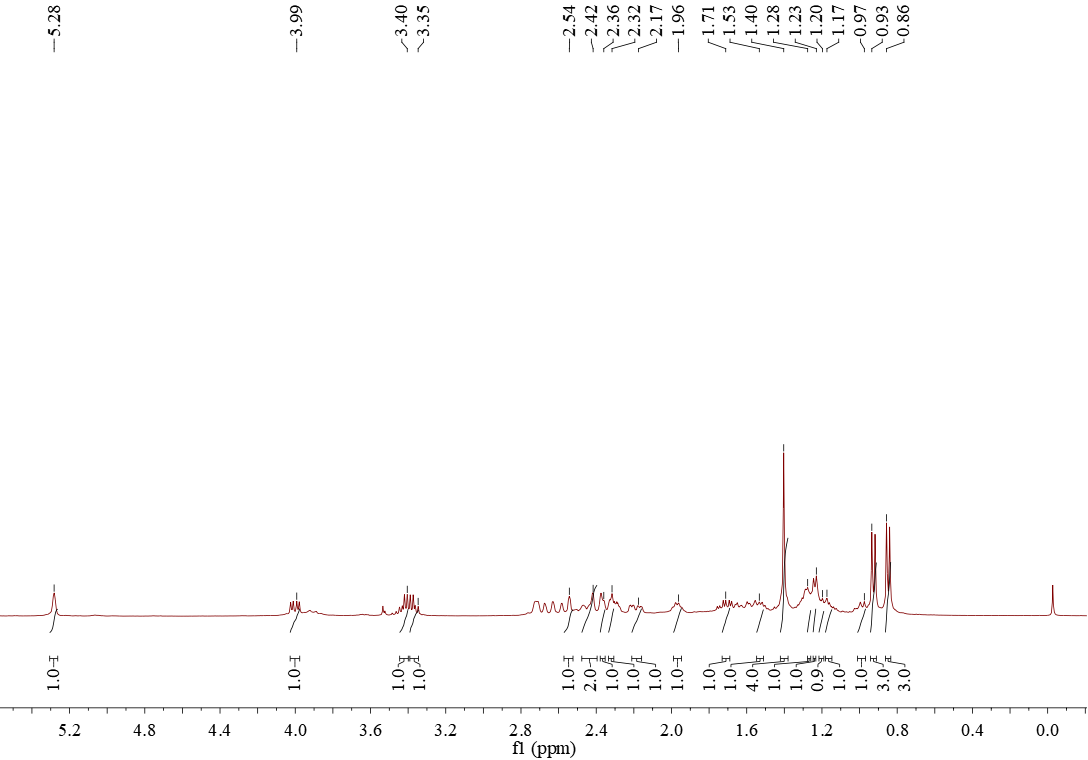


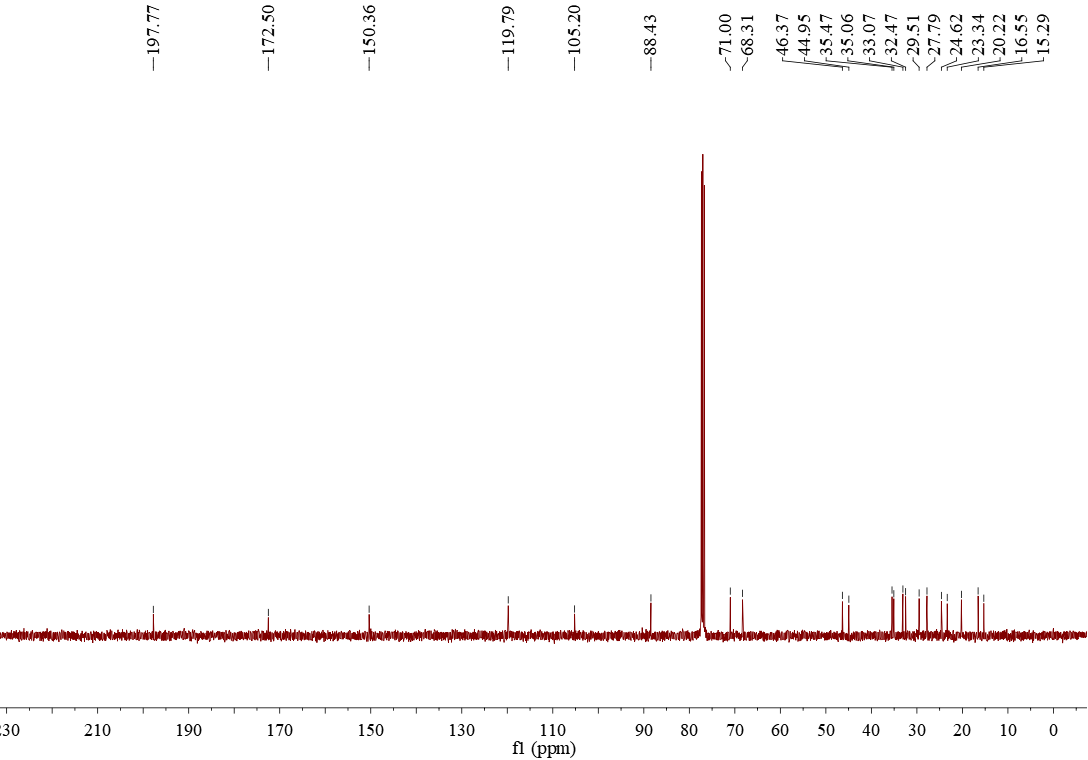


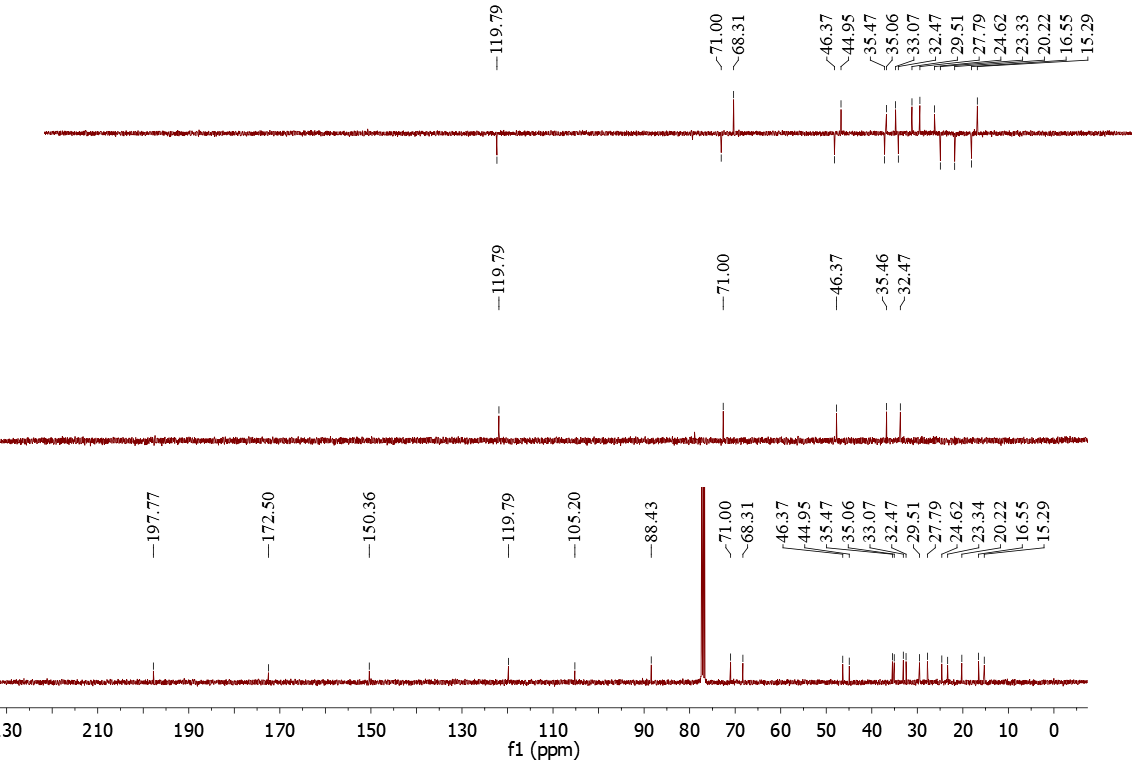


Supplementary Figure 4. The NMR data of compound 4 in CDCl_3_ (500 MHz for ^1^ H NMR, 125 MHz for ^13^ C NMR)


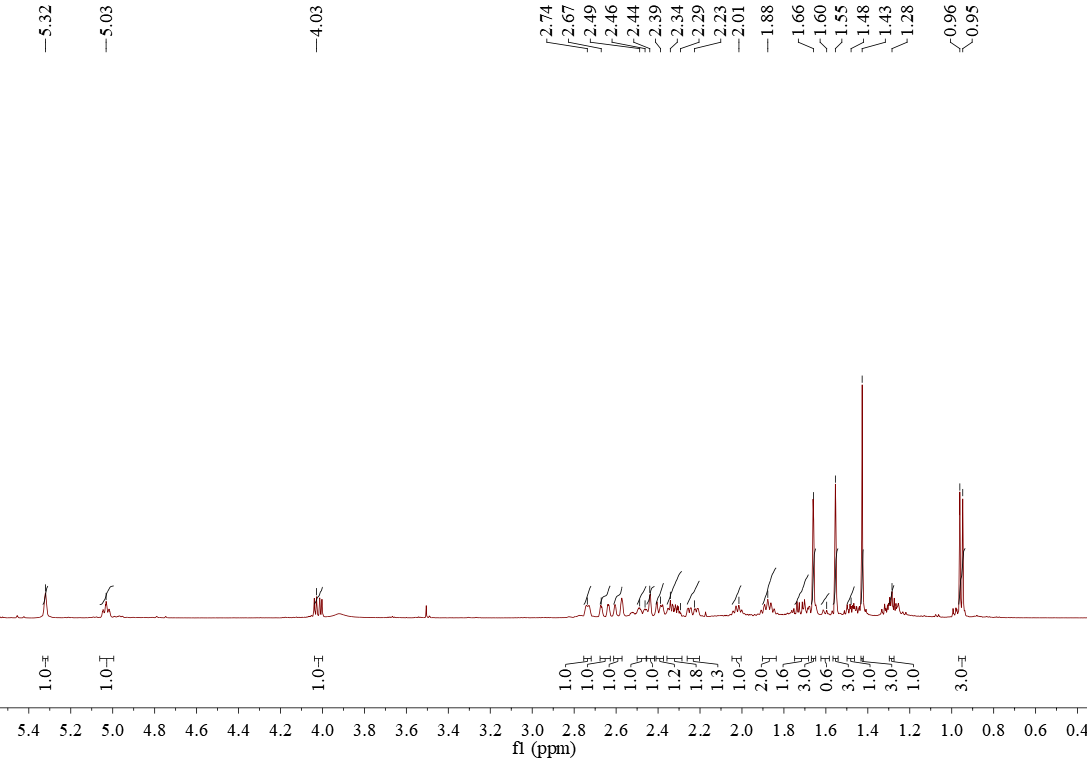

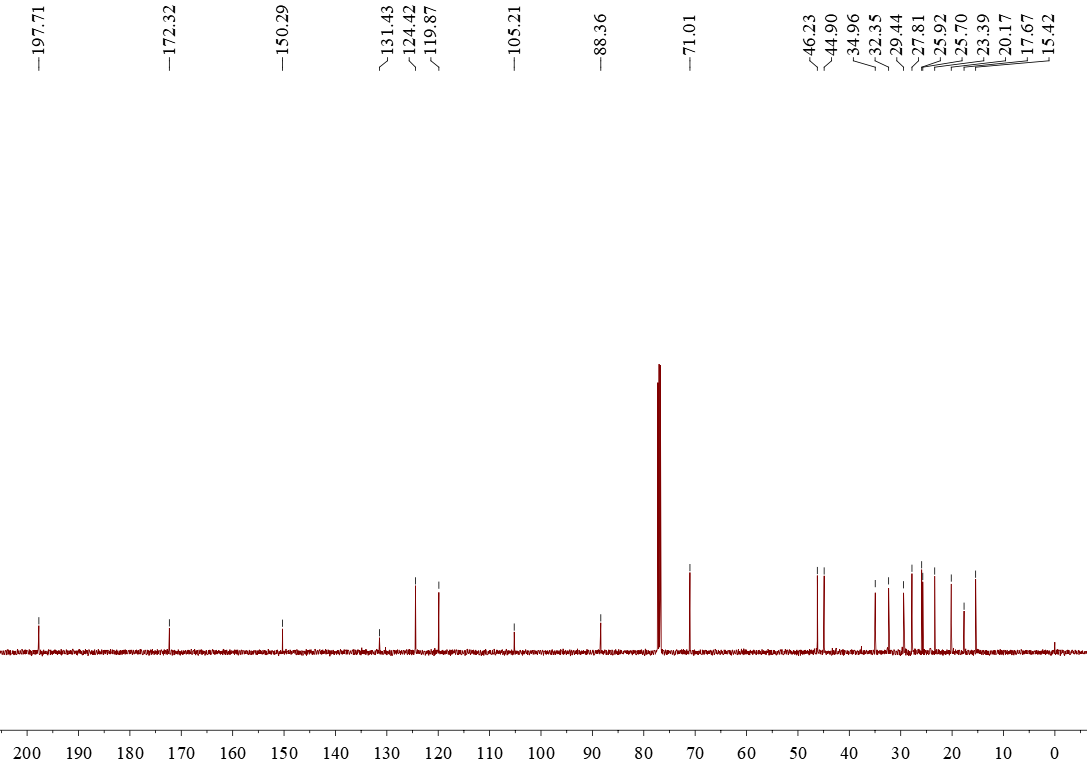

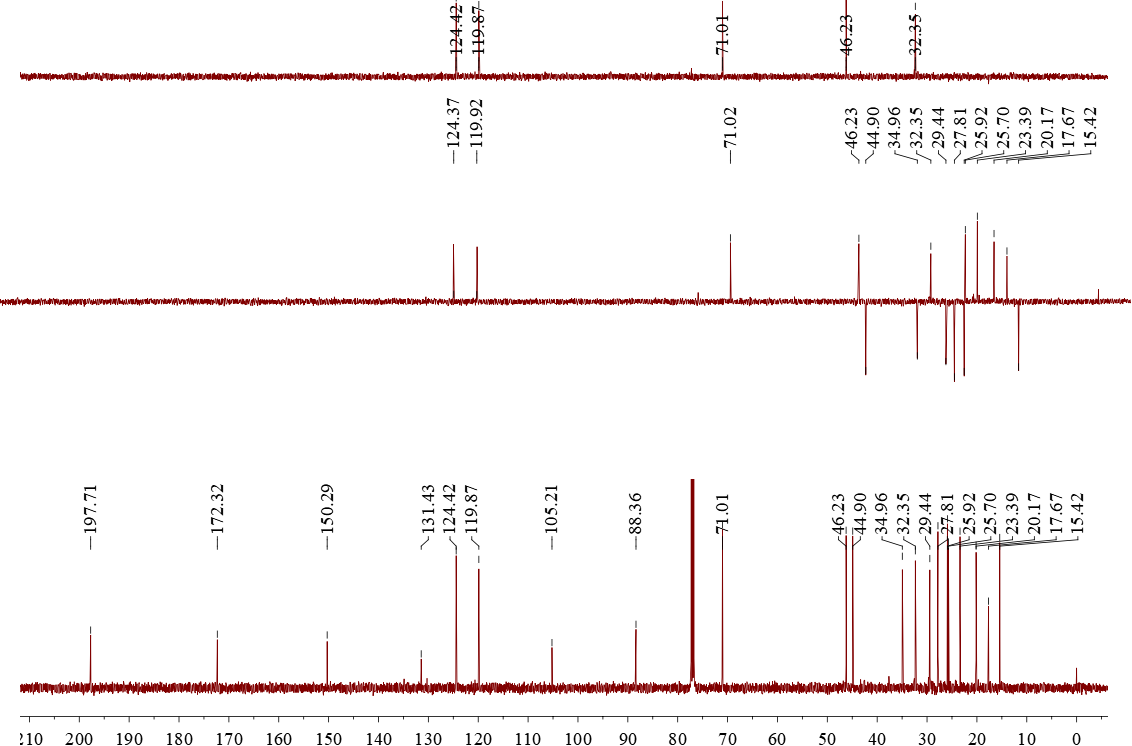


Supplementary Figure 5. The NMR data of compound 5 in CDCl_3_ (500 MHz for ^1^ H NMR, 125 MHz for ^13^ C NMR)


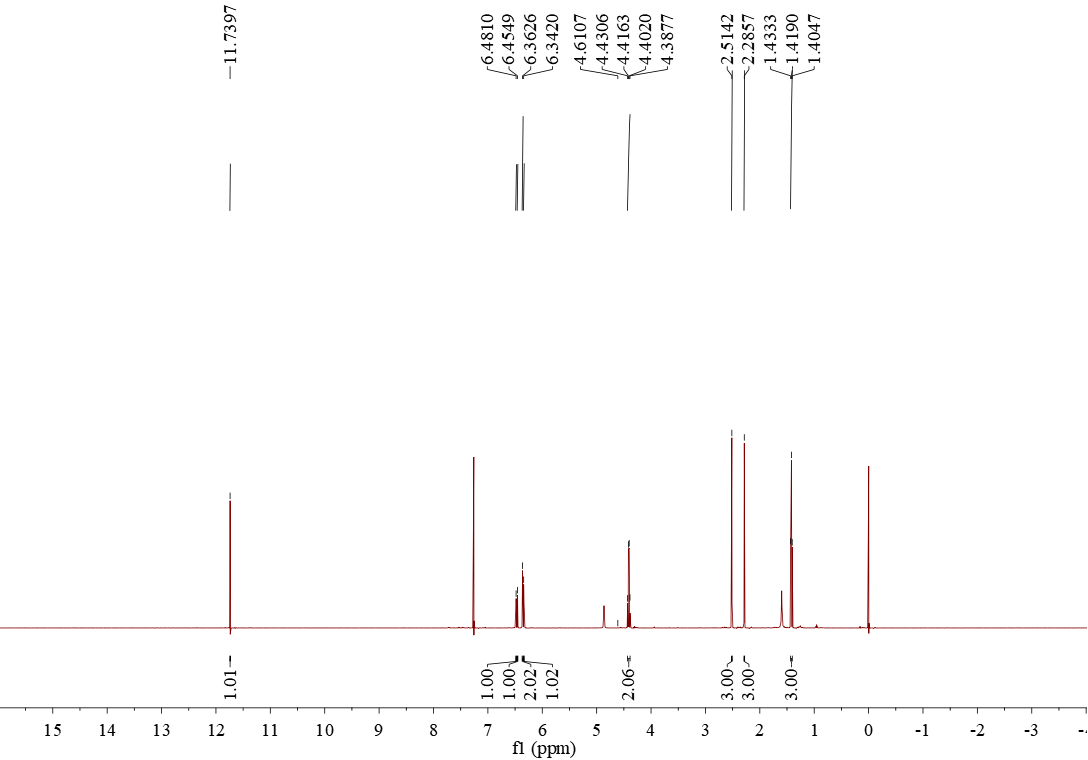


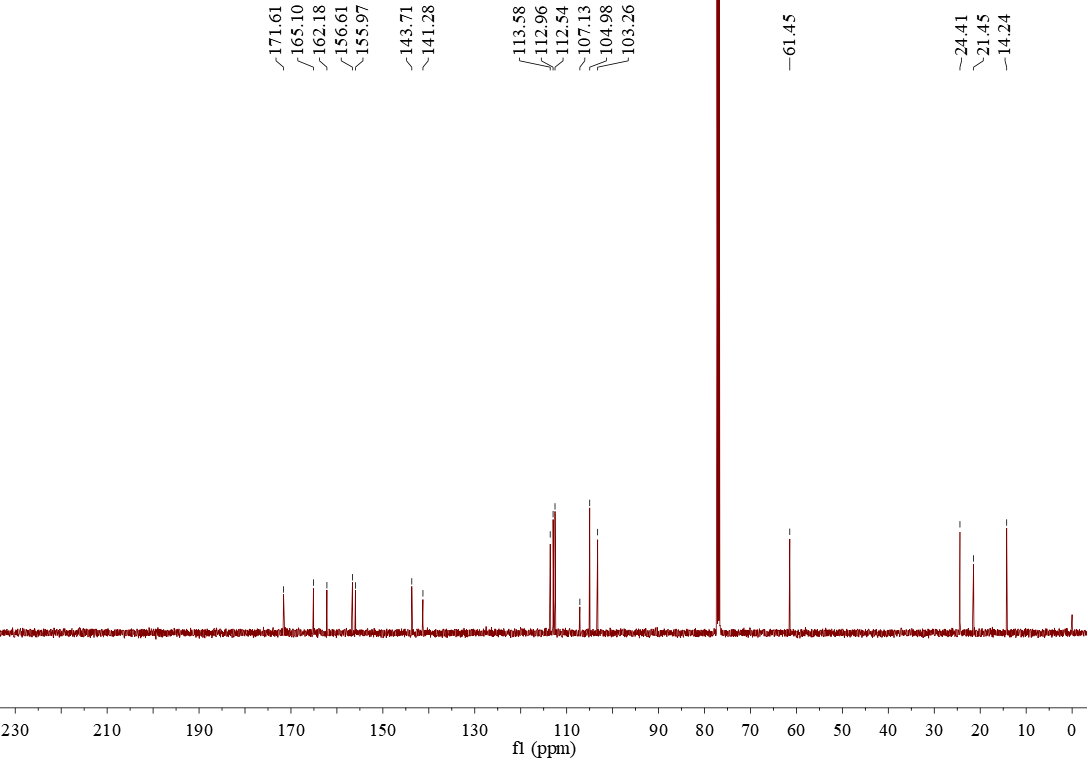

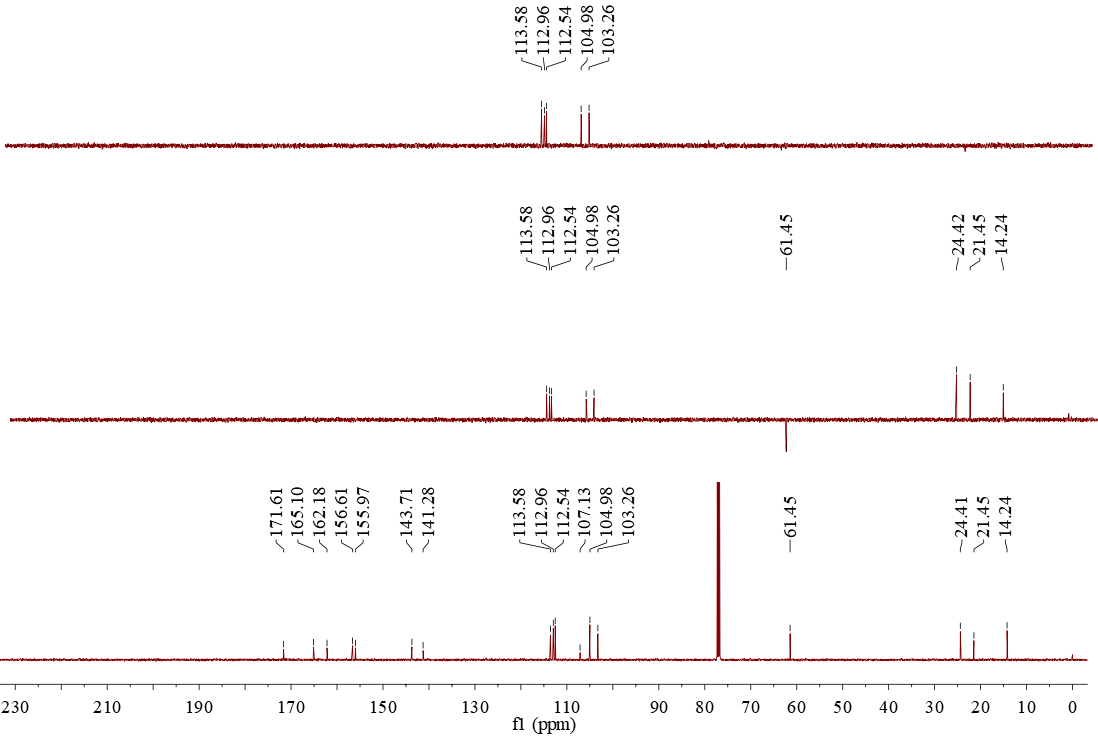


Supplementary Figure 6. The NMR data of compound 6 in DMSO-d_6_ (500 MHz for ^1^ H NMR, 125 MHz for ^13^ C NMR)


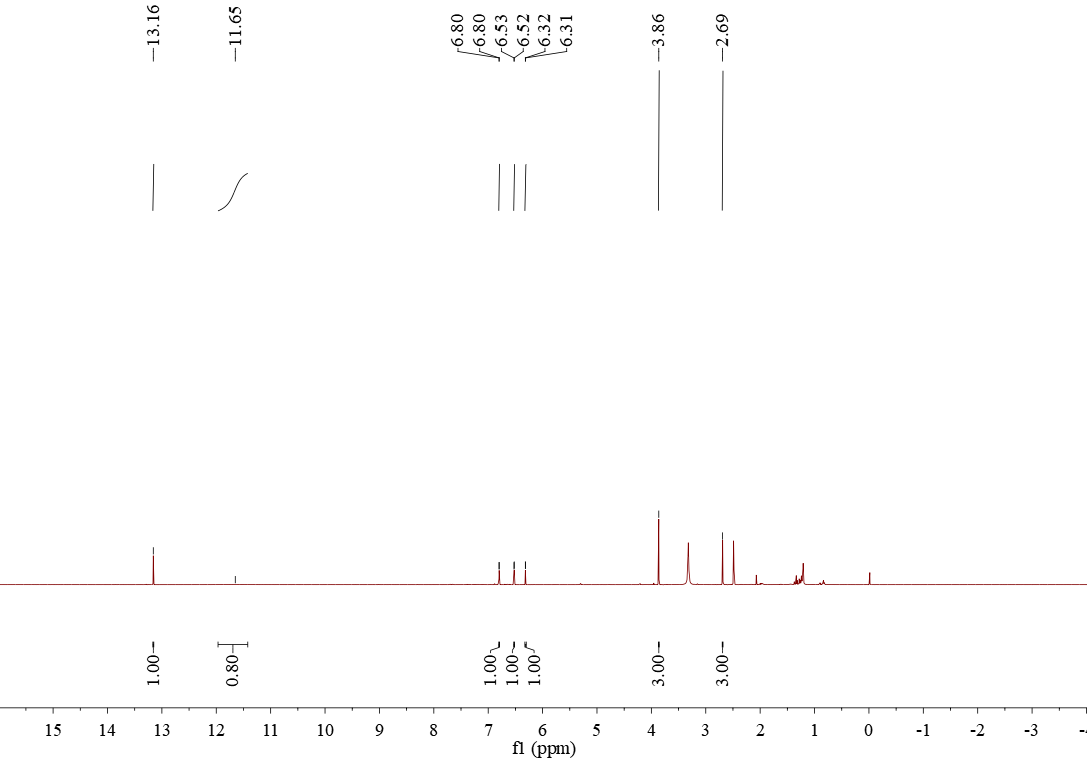


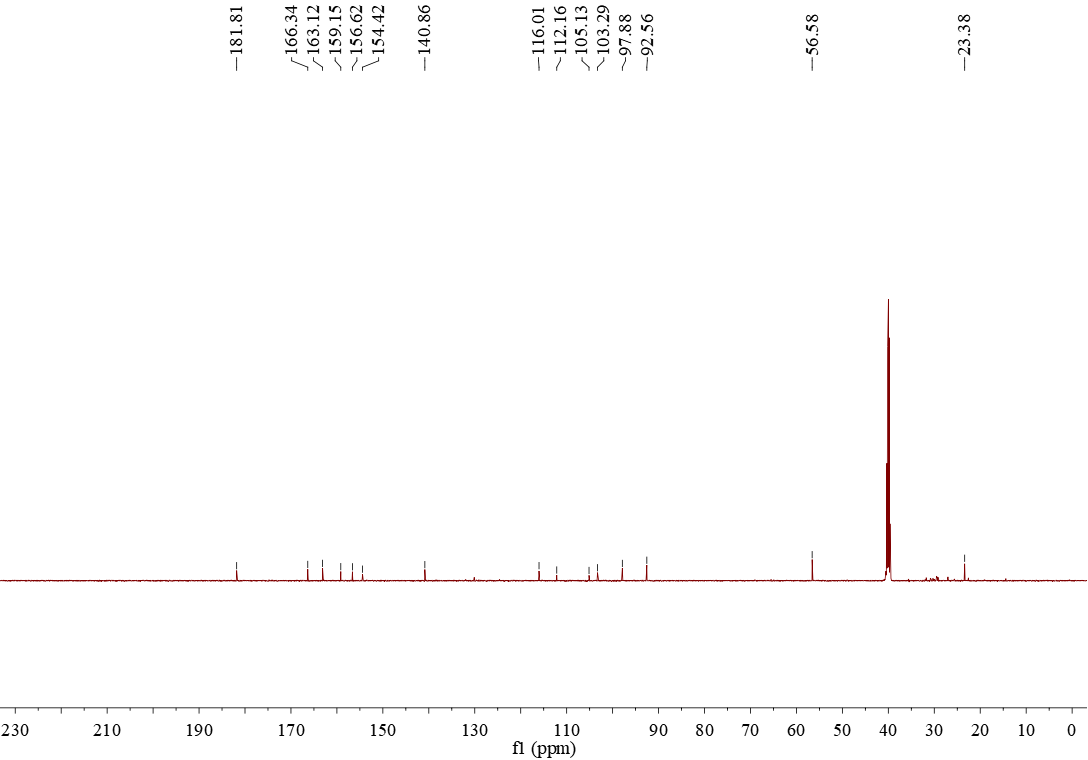


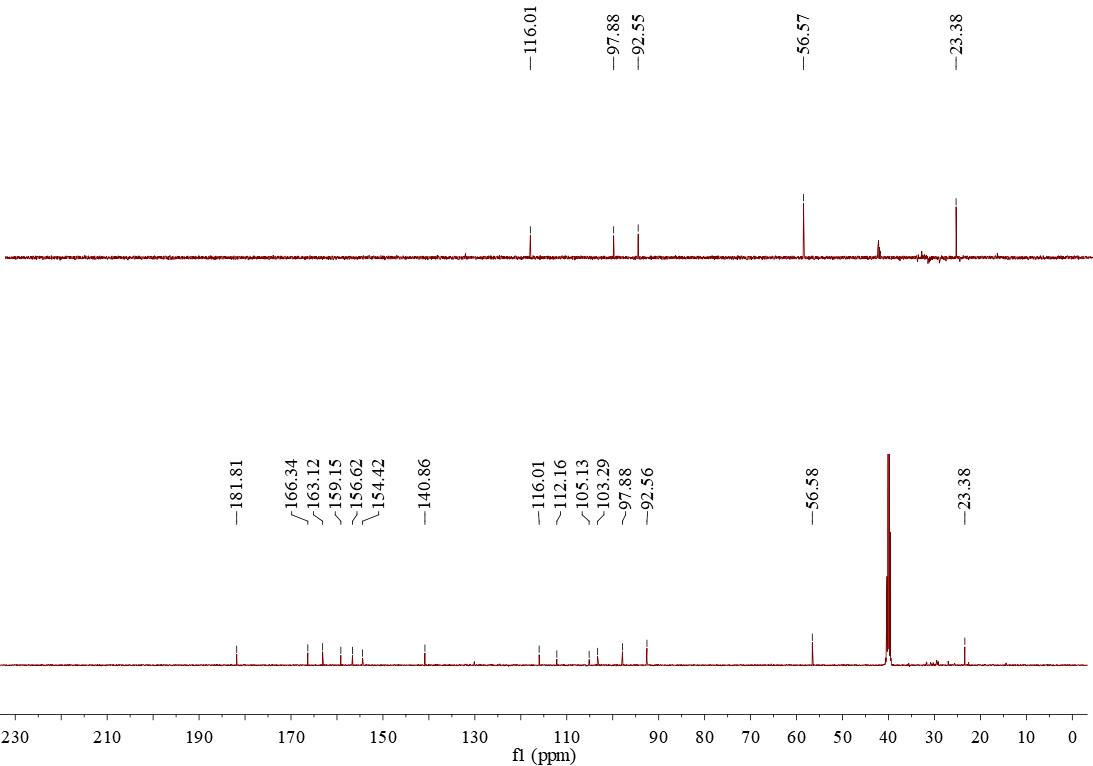


Supplementary Figure 7. The minimum inhibitory concentration (MIC, µg/mL) of the monomeric compounds (1-6) against *S. agalactiae* GBS-1 **(A)**, *S. aureus* SA-1 **(B)** and *E. coli* EC-1 **(C)** respectively.


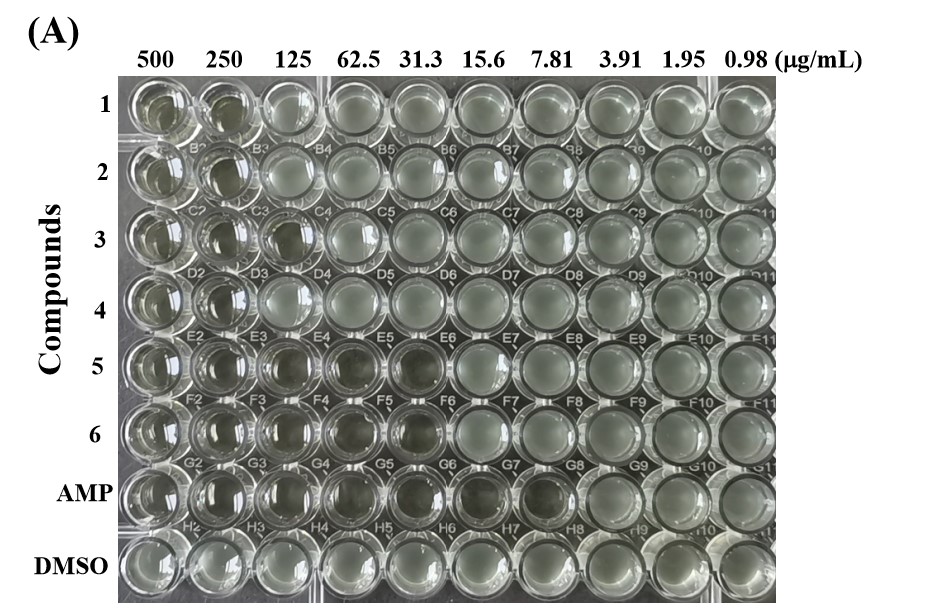


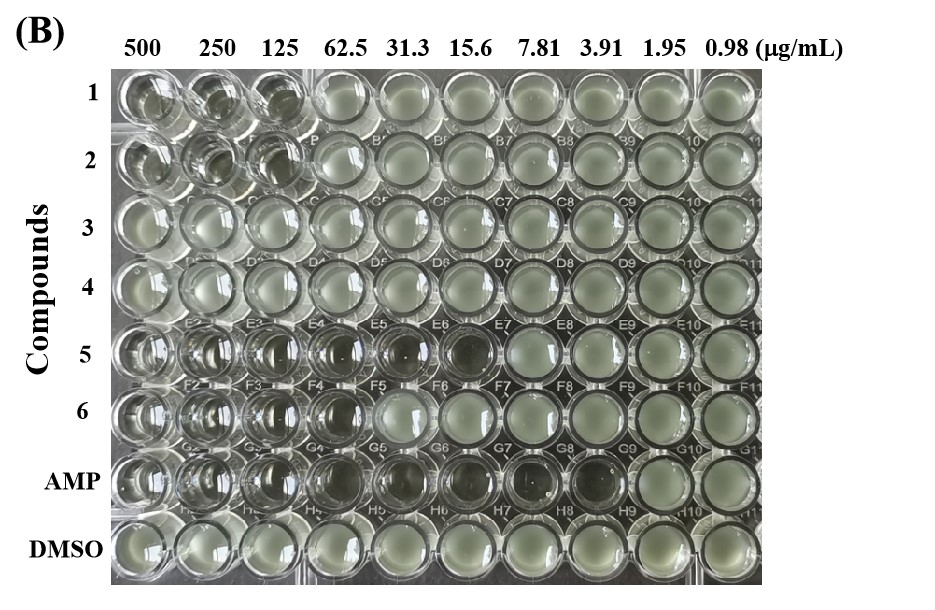


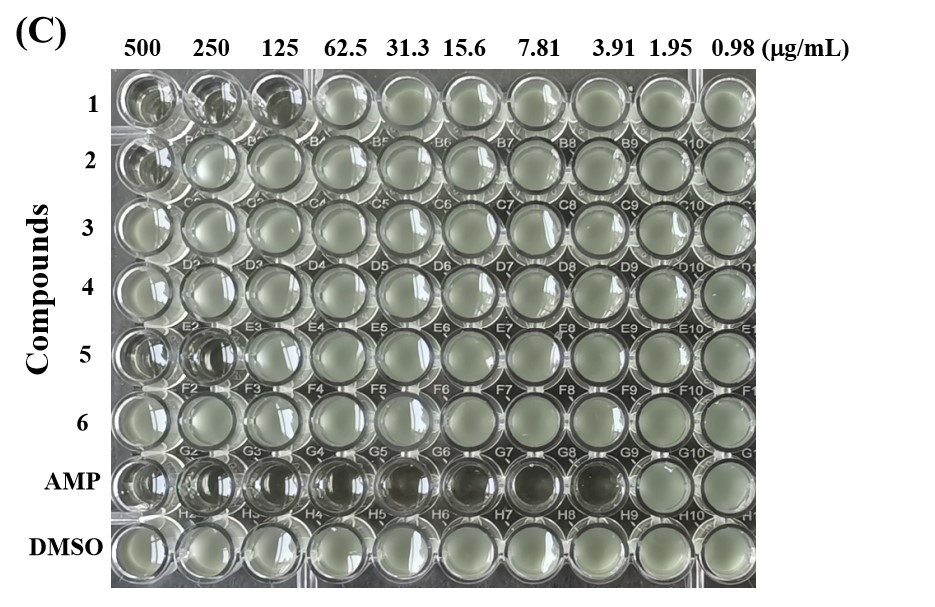

Supplement: Supplemental Information 2 [file peerj-11-15461-s002.docx]
